# Cortical information flow during inferences of agency

**DOI:** 10.3389/fnhum.2014.00609

**Published:** 2014-08-14

**Authors:** Myrthel Dogge, Dennis Hofman, Maria Boersma, H. Chris Dijkerman, Henk Aarts

**Affiliations:** ^1^Department of Psychiatry, University Medical Center UtrechtUtrecht, Netherlands; ^2^Department of Psychology, Utrecht UniversityUtrecht, Netherlands

**Keywords:** sense of agency, inferences, phase synchronization, EEG, connectivity, goal-directed processes, outcome priming

## Abstract

Building on the recent finding that agency experiences do not merely rely on sensorimotor information but also on cognitive cues, this exploratory study uses electroencephalographic recordings to examine functional connectivity during agency inference processing in a setting where action and outcome are independent. Participants completed a computerized task in which they pressed a button followed by one of two color words (red or blue) and rated their experienced agency over producing the color. Before executing the action, a matching or mismatching color word was pre-activated by explicitly instructing participants to produce the color (goal condition) or by briefly presenting the color word (prime condition). In both conditions, experienced agency was higher in matching vs. mismatching trials. Furthermore, increased electroencephalography (EEG)-based connectivity strength was observed between parietal and frontal nodes and within the (pre)frontal cortex when color-outcomes matched with goals and participants reported high agency. This pattern of increased connectivity was not identified in trials where outcomes were pre-activated through primes. These results suggest that different connections are involved in the experience and in the loss of agency, as well as in inferences of agency resulting from different types of pre-activation. Moreover, the findings provide novel support for the involvement of a fronto-parietal network in agency inferences.

## Introduction

Humans generally feel in control of their actions and the events that follow from them. This sense of agency plays a key role in self-awareness as well as social interaction (Haggard and Tsakiris, 2009; Ruys and Aarts, 2012). Although experiences of self-agency arise naturally in most individuals, abnormalities in agency processing, such as feeling in control over externally generated outcomes, or, oppositely, experiencing a loss of control over outcomes that one did produce, have been observed in a variety of psychiatric and neurological disorders (Blakemore et al., 2002). Examining the neural substrates underlying self-agency in the healthy brain thus is an important step to comprehend the origin of disturbed agency experiences, and eventually uncover possible ways to alleviate them.

The experience of agency has primarily been studied from the perspective of comparator models that are part of the motor control system (Frith et al., 2000). These models often rely on paradigms in which visual, tactile, or auditory feedback of the participants' action is manipulated (e.g., Sperduti et al., 2011; David, 2012). According to the comparator model, the execution of an action is accompanied by the prediction of sensory action-outcomes based on internal copies of movement-predicting signals (i.e., efference copies) generated by the motor system. Because internal motor predictions are generally fast and reliable, sensory outcomes are readily perceived as self-produced when these predictions correspond with the actual outcome (Frith et al., 2000). This motor prediction process of agency has been found to be associated with brain activity in various areas, including the superior temporal gyrus, the inferior parietal lobe, as well as motor regions such as the pre-supplementary motor area and the cerebellum (for an overview, see Sperduti et al., 2011).

According to the comparator model, experiences of agency are less likely to occur when the motor system cannot produce an efference copy (i.e., when acts are not self-generated) or when these signals are weak or noisy, such as when there is no clear causal relationship between an action and an effect. However, recent research has demonstrated that people can feel in control over externally generated events (Wegner et al., 2004) and in the absence of high action-effect contingency (Moore et al., 2009; Van der Weiden et al., 2011). These findings strongly suggest that agency experiences can also emerge via a different route. This alternative route, specified by the inference model, involves cognitive inferences of the correspondence between action outcomes and prior activation of information about the outcome (Wegner, 2002). Despite the role of these inference processes in the emergence of agency (Moore et al., 2009; Sato, 2009; see also Synofzik et al., 2008, 2013), their neural basis has hitherto received relatively little empirical attention. Another issue that remains unclear from prior work is how brain regions associated with agency interact and exert influence over each other. The present study builds on recent advancement in the quantification of neural communication to examine the interactions between cortical regions during inferences of agency.

### Agency inferences

The inference model proposes that upon observing an event, people determine whether or not it has resulted from their actions by comparing the outcome with prior activated information or thoughts about action-effects. If there is a match, they ascribe the action-outcome to themselves, whereas, in case of a mismatch, the effect is ascribed to an external cause. Although this account involves a predictive element regarding action-outcomes similar to the comparator model, the prior expectations specified in this model only minimally depend on motor signals. Instead, these expectations pertain to cognitive priors such as intentions and beliefs (Synofzik et al., 2013). Moreover, even though predictive elements are involved in inference processes, the critical information is provided by the action outcome (Synofzik et al., 2013).

It is important to note that inferences of agency are normally thought to result from intentions. That is, if an intention to produce a certain outcome matches the actual sensory consequences following one's action, people tend to experience causal responsibility for these consequences, whereas if the intention mismatches with the observed outcome, a reduced sense of agency is experienced (Wegner, 2002). Intriguingly, however, recent research suggests that prior knowledge regarding action outcomes does not necessarily need to be explicitly activated for agency inferences to occur, but can also consist of outcome primes as a source of agency (Aarts et al., 2005; Linser and Goschke, 2007; Jones et al., 2008; Belayachi and van der Linden, 2010; Dannenberg et al., 2012; Ruys and Aarts, 2012). This evidence possibly accounts for the emergence of experienced agency in everyday situations where people do not produce action-outcomes themselves or lack awareness of the actual causes of their behavior.

Although goals and primes give rise to similar inferences of agency, there is some preliminary evidence to suggest that the two sources produce qualitatively different effects (Van der Weiden et al., 2013). Specifically, pursuing a goal instigates a control process that causes people to focus on the specific outcome one wants to reach and, at the same time, to inhibit all other possible outcomes (Fishbach and Ferguson, 2007; Förster et al., 2007; Aarts, 2012). Consequently, inference processes based on goals are very specific and reliable in the detection of deviations from intended outcomes. These goal-directed control processes are less likely to occur in case of outcome priming, because outcome priming is assumed to merely enhance the accessibility of the outcome representations and other information associated with it (Van der Weiden et al., 2013). This implies that agency inferences based on priming are less sensitive to deviations and hence have a noisier processing mechanism than goal-directed processes (Van der Weiden et al., 2013). Based on these qualitative differences between inferences resulting from goals and primes, we not only examined the neural communication between cortical regions underlying goal-based inferences but, for exploratory purposes, also investigated these neural processes during prime-based inferences.

### Neural communication and agency inferences

Cognitive functioning, including inferences of agency, is dependent on the integration of information within and between functionally specialized brain sites (Varela et al., 2001; Stam and van Straaten, 2012a). There is increasing agreement that this integration, or more precisely, the communication between neurons, arises from synchronization of neural activity (Salinas and Sejnowski, 2001; Varela et al., 2001; Buzsáki and Draguhn, 2004; Fries, 2005; Schnitzler and Gross, 2005; Sauseng and Klimesch, 2008). Specifically, neurons' responsiveness has the property to oscillate, referring to fluctuations in excitability of their membrane potential (Buzsáki and Draguhn, 2004). These fluctuations create time windows in which a neuron is most responsive to signals by other neurons (Buzsáki and Draguhn, 2004). Hence, for two neurons to successfully exchange information, their excitability period needs to be aligned, which happens whenever they oscillate in phase (Buzsáki and Draguhn, 2004; Fries, 2005). In contrast, when phase synchronization between the oscillations of two neurons is absent, their communication is inhibited (Fries, 2005). Accordingly, the neural networks underlying agency processing can be studied by examining the synchronization of neural activity, and thus the exchange of information between local and distant groups of neurons (Varela et al., 2001).

Two recent studies using functional magnetic resonance imaging (fMRI) have provided some insights into the neural networks underlying agency processing. In one study, participants were asked to indicate perceived control over actions based on congruent or incongruent movement feedback (David et al., 2007). Increased connectivity was observed between the pre-motor cortex, cerebellum, and posterior parietal cortex (PPC) when movements were correctly identified as externally generated, and between the insula and somatosensory cortex when movements were correctly classified as self-generated (David et al., 2007; David, 2012). In another study, leading and lagging networks were identified during experiences of loss of control in response to incongruent visual feedback (Nahab et al., 2011). The leading network consisted, among others, of the inferior parietal lobe and the insula and was shown to send information to a lagging network consisting of several areas in the posterior parietal and prefrontal lobe. The authors interpreted the leading network as being involved in the comparison of motor predictions with actual effects, whereas the lagging network (in particular the prefrontal lobe) was thought to be responsible for the translation of the outcome of this comparison into higher order processing of agency, such as the conscious awareness of this experience.

Although the aforementioned connectivity studies provide a first glimpse into neural networks underlying experiences of agency and to the direction of information flow between them, they deal with agency processes informed by motor predictive signals, and not by cognitive inferences processes *per se*. A recent fMRI study addressed this notion by examining the neural substrates of goal-based agency inferences (Renes et al., 2013). During the ascription of outcomes to oneself, activation was observed in the inferior parietal lobe, the superior frontal cortex and the medial prefrontal cortex, implying that the lagging network identified by Nahab et al. (2011) might indeed be involved in the previously mentioned higher order agency processing.

### The present study

In the present study we further examine and extend these findings by analyzing the pattern of information flow during inferences of agency using measures of (directed) phase synchronization. By doing so, we not only build on recent calls for a shift from localization to network perspectives on agency processing (David, 2012), but also expand prior work on the connectivity underlying the sense of agency by employing a more direct measure of neural communication.

To explore the cortical interactions underlying inferences of agency we used an action-outcome task in which participants perform an action (pressing a key) that is followed by a sensory effect (the color word red or blue presented on the computer screen) that either matches or mismatches with pre-activated knowledge of this outcome. After observing the outcome, self-agency over producing the outcome is reported. Importantly, participants learn that the outcome they observe is not always caused by their actions but can be determined by the computer as well. As a consequence, sensorimotor predictive processes are unreliable in this task, allowing us to pinpoint agency experiences that are informed by inferences. Furthermore, pre-activation of knowledge about outcomes in manipulated by explicitly instructed goals to produce the outcome or by briefly presented primes of the outcome, thus allowing us to study goal-based and prime-based agency inferences.

To examine the neural communication of agency inferences we used the electroencephalogram (EEG), which has a temporal resolution that is sufficient to non-invasively examine phase synchronization (Sauseng and Klimesch, 2008; Stam and van Straaten, 2012a). Based on prior work we are particularly interested in coupling strength between parietal and frontal regions and the direction of information flow between them.

## Methods

### Participants

Thirty right-handed participants (*M*_age_ = 21.03, *SD*_age_ = 3.20; 22 females) who indicated no current neurological condition, mental illness or use of psychiatric medication took part in the experiment. Participants were asked to refrain from the consumption of caffeine 3 hours prior to the experiment. All participants received course credit or a monetary reward in exchange for their participation. The study received approval from our internal faculty board (Social and Behavioral Sciences) at Utrecht University. Furthermore, written informed consent of each participant was obtained.

### Agency inference task

The agency inference task was adapted from Renes and Aarts (in preparation). Similar to playing a slot machine, this task required participants to stop a sequence of rapidly presented information to produce a particular outcome (i.e., the color word red or blue) on the computer screen. Specifically, participants pressed a key in response to a cue while viewing alternating letter strings. Upon pressing this key, the stream of letter strings stopped and the color word “red” or “blue” was presented. This outcome could either match or mismatch with prior knowledge regarding the action-effect (i.e., goals or outcome primes; see below). In addition, participants learned that the computer could have caused the presented outcome as well. In other words, the cause of the observed effect was ambiguous (Aarts et al., 2005; Sato, 2009). After viewing the sensory effect following their key press, participants reported experienced agency over causing the perceived effect.

**Figure 1:**
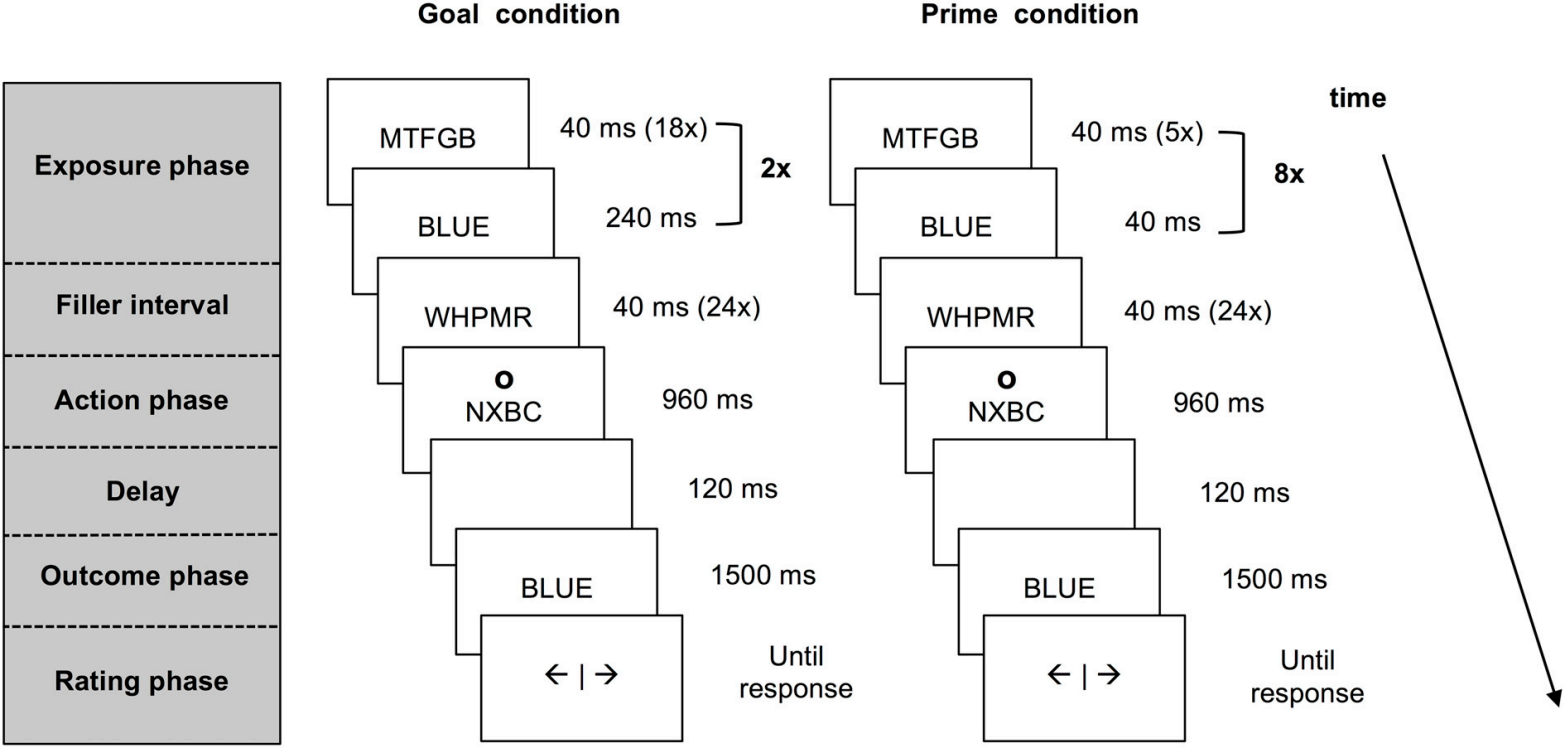
**Schematic presentation of a match trial in the agency inference task for the goal condition and the prime condition**. Both goal and prime trials start with the pre-activation of a color word that is presented within a stream of letter strings. In the goal trials participants are instructed to produce this outcome. In the prime trials participants are merely exposed to the prime words. After a short interval participants press a key in response to an action cue appearing above or below the letter strings. Upon this key press the stream of information stops and a color word matching or mismatching the pre-activated word is presented. Participants are asked to report experienced self-agency over this outcome.

Each trial consisted of five different phases: an exposure phase, a filler interval, an action phase, an outcome phase and a rating phase (see Figure 1). The last four phases were identical for all trials. During the filler interval, participants attended to rapidly alternating letter strings. This interval served as a delay between exposure to pre-activated information and the action that was also present in previous work on agency inferences (e.g., Van der Weiden et al., 2013). In the action phase, participants responded to a circle (the letter “o” presented in Arial 24 pt. at an approximate visual angle of 2.10°) that appeared above or below the letter strings, by pressing the corresponding upper or lower key on a response box with their right index finger. This action cue was included to ensure that participants paid attention to the outcome prime or goal presented amidst of the letter strings. The interval in which a response could be given lasted 800 ms. If participants pressed the key within this interval, the strings continued to alternate until the end of a 960 ms lasting interval, whereas if they pressed too late, an error message occurred and the trial was processed as missing.

Following the action phase, the color word “red” or “blue” (counterbalanced between trials) was shown for 1500 ms, after a short delay of 120 ms. To ensure that participants would maintain looking at the letter strings, participants were told that pressing the key during the presentation of a string containing the letter R (e.g., MWRT) would cause the word “red” to appear, whereas a key press during the presentation of a string containing the letter “B” (e.g., BTSZW) was followed by the word “blue.” In reality, the computer determined the presentation of color words.

After each trial, experienced agency was assessed during a rating phase by asking participants to what extent they felt their key press caused the presented color word to occur. They could respond by moving a square on a 9-point analog scale ranging from the Dutch word “*niet*” (in this context roughly corresponding to: “Not at all” to “*wel*” (“Very much”). The square had to be moved at least one position to the left or the right of the scale, starting in the center (i.e., answer “5”). This caused the data to consist of split responses (i.e., data ranging from 1 to 4 and 6 to 9). In order to form a continuous scale ranging from 1 to 8, the agency ratings were recoded (i.e., 9 = 8, 8 = 7, 7 = 6, and 6 = 5).

#### Pre-activated knowledge about outcomes

As mentioned earlier, the exposure phase was not identical for all trials. Specifically, in this phase knowledge regarding the outcome was activated by either goals or by primes.

In goal trials, participants were exposed to a series of letter strings followed by a color word that was clearly presented on the screen for 240 ms. This sequence was repeated twice (see Figure 1), using the same color word. Participants were instructed to form the goal to produce the color word that appeared within the series of letter strings.

In outcome prime trials, participants viewed five random letter strings followed by a briefly presented color word (40 ms). This sequence of events was repeated eight times, resulting in a total of eight identical primes during a 1920 ms period (see Figure 1). Importantly, participants were not instructed to formulate a goal in the prime trials.

Note that, in contrast to prior studies (Van der Weiden et al., 2013), the duration and moment of the exposure phase was identical for both types of pre-activated outcome information. Accordingly, differences between prime and goal based inferences could be examined in a more controlled manner.

The goal trials and outcome prime trials were presented in two separated blocks which each consisted of 64 randomly presented trials. All participants started with the prime condition to prevent transference of instructions from the goal condition to the prime condition (i.e., to prevent participants from using the primed information to form a goal). In half of the trials, pre-activated color words corresponded with the actual outcome, whereas in the other half of the trials they did not correspond with this outcome. Participants practiced for both blocks before the onset of the experiment (eight trials per practice block). After completing these practice trials participants completed the outcome-priming block, followed by a practice block for the goal condition (four trials) and the actual goal block. In between the two blocks participants were allowed to have a break. In addition, participants paused for 30 s after completing the first half (i.e., 32 trials) of each block.

### EEG recording and pre-processing

EEG was recorded at a sampling rate of 2048 Hz during the entire agency inference task from 32 electrodes positioned according to the international 10/20 system using the BioSemi Active Two EEG system (BioSemi). The Electro-oculogram (EOG) was measured from electrodes placed on the suborbit and supraorbit of the right eye and on the outer canthi of both eyes. Raw EEG data was band pass filtered offline (0.5–50 Hz) with a roll-off of 48 dB/oct and a 50 Hz Notch filter. Time series were re-referenced against an average reference. In order to correct for eye movements, Gratton and Cole's method (Gratton et al., 1983) was used. A semi-automated artifact correction tool (Brain Vision Analyzer software package; Version 2.0), allowing a maximum difference of 50 μ V/ms, was employed to detect further artifacts. The corrected data was chunked down to 128 trial-specific segments that started at the onset of the outcome presentation and ended after 1000 ms. This time window corresponds to the interval of interest used in prior work on the neural basis of agency inferences (Renes et al., 2013).

### Functional connectivity

EEG was employed to assess both bidirectional and directional neural communication during agency inferences; quantified by the phase lag index (PLI; Stam et al., 2007) and directed phase lag index (dPLI; Stam and van Straaten, 2012b) respectively.

#### Phase lag index

The PLI identifies statistical interdependency of two time series based on the level of asymmetry of the distribution of their phase differences (for mathematical details see Stam et al., 2007). Since the PLI only reflects correlations between signals of which the phase difference deviates from zero, it is less affected by common source problems and amplitude changes than other connectivity measures (however, see Muthukumaraswamy and Singh, 2011). The PLI ranges from 0 to 1, with a score of zero indicating no coupling or coupling that might result from common source problems, and a score of 1 indicating perfect coupling.

BRAINWAVE software (version 9.75) was used to compute the instantaneous phase (using a Hilbert transformation) and PLI between all pairs of electrodes for each trial in the broadband (2–50 Hz), delta band (2–4 Hz), theta band (4–8 Hz), alpha band (8–12 Hz), beta band (13–30 Hz), and gamma band (30–40 Hz). By doing so, trial specific 32 × 32 connectivity matrices were created. Given that the present study aims to examine functional connectivity associated with agency experiences emerging from inferences, rather than connectivity as a function of task conditions, we decided to examine the low vs. high agency contrast within each task condition (i.e., as a function of matching and type of pre-activation). In line with prior research (Renes et al., 2013), the aforementioned 32 × 32 trial matrices were sorted into two groups based on agency ratings (Low agency: rating ≤ 4, High agency: rating ≥ 5). The frequency distributions of agency ratings for matching and pre-activation cells are presented in Supplementary Figure S1. To allow for group comparison, average matrices were created for each possible combination of type of pre-activation, matching and level of agency. This resulted in eight average 3D group matrices comprising PLI values for all possible pairs of electrodes per participant.

Nonparametric permutation tests adapted from Boersma et al. (2013) were used to test for differences in PLI for all possible electrode pairs between low agency and high agency for match and mismatching conditions (i.e., low agency vs. high agency for goals matching the outcome, low agency vs. high agency for goals mismatching the outcome, low agency vs. high agency for primes matching the outcome and low agency vs. high agency for primes mismatching the outcome). These tests involved a resampling method with replacement, which was used to generate ten thousand random pairs of groups from the two originally specified observations (i.e., low and high agency), across participants^1^. By comparing the mean PLI values for all electrode pairs between these random groups, a distribution of differences for all pairwise connections was created. The position of the original difference value in this distribution was used to determine *p*-values for each contrast. Significant differences (alpha = 0.05) were visualized using a modified version of the topoplot function in the EEGlab toolbox (Delorme and Makeig, 2004). Specifically, networks were plotted in which each node is represented by an EEG electrode and the links between the nodes correspond to a significant difference in connectivity between low and high agency.

#### Directed phase lag index

dPLIs were calculated to examine the direction of information flow of pairwise connections. Similar to the PLI the dPLI is a measure of the asymmetry of the distribution of phase differences of two signals (Stam and van Straaten, 2012b). However, dPLI also assesses the direction of the asymmetry (i.e., the probability that the phase of the signal measured at electrode X is smaller than the phase of the signal measured at electrode Y), whereas PLI merely determines the presence of absolute asymmetry. The direction of the asymmetry allows one to infer whether a signal recorded from a node is phase leading (i.e., sending information) or phase lagging (i.e., receiving information) compared to the signal recorded from all the other nodes (Stam and van Straaten, 2012b). Specifically, time series measured from a node with a dPLI score larger than 0.5 are thought to be leading in phase, whereas a dPLI score smaller than 0.5 indicates the opposite pattern (Stam and van Straaten, 2012b). In the present study a modified version of the BRAINWAVE software (version 9.70) was used to assess directional connectivity between pairs of nodes.

For each trial, dPLI matrices for all electrode pairs and average group matrices corresponding to each possible combination of matching, type of pre-activation and agency were constructed. These average matrices were used to obtain dPLI values for all participants for each connection that significantly differed in PLI between groups. Exploratory one-sample *t*-tests were used to examine whether dPLI values of the connections significantly differed from 0.5. Corrections for multiple comparisons were made by means of Benjamini and Hochberg's (1995) false discovery rate procedure. These analyses were performed using SPSS (version 20).

## Results

### Behavioral data

#### Agency ratings

One hundred and eleven trials (2.89% of the total amount) were excluded from the analyses due to the absence of a key press within the interval of the action phase. Mean agency ratings were calculated for matches and mismatches in the goal trials and in the prime trials. Visual inspection of the data as well as normality tests indicated non-normality of the data. However, considering the robustness of ANOVA for these departures from normality, we refrained from the use of non-parametric alternatives. The mean ratings were submitted to a 2 (type of pre-activation: goal vs. prime) × 2 (matching: mismatch vs. match) repeated measures ANOVA. This analysis yielded a main effect of matching, *F*_(1, 29)_ = 13.06, *p* = 0.001, η^2^_ρ_ = 0.31, indicating higher agency experiences when pre-activated outcome information was consistent as opposed to inconsistent with the actual outcome. Moreover, an interaction between type of pre-activation and matching was observed, *F*_(1, 29)_ = 5.39, *p* = 0.03, η^2^_ρ_ = 0.16. The main effect for type of pre-activation was not significant, *F*_(1, 29)_ = 1.25, *p* = 0.27, η^2^_ρ_ = 0.04.

**Figure 2:**
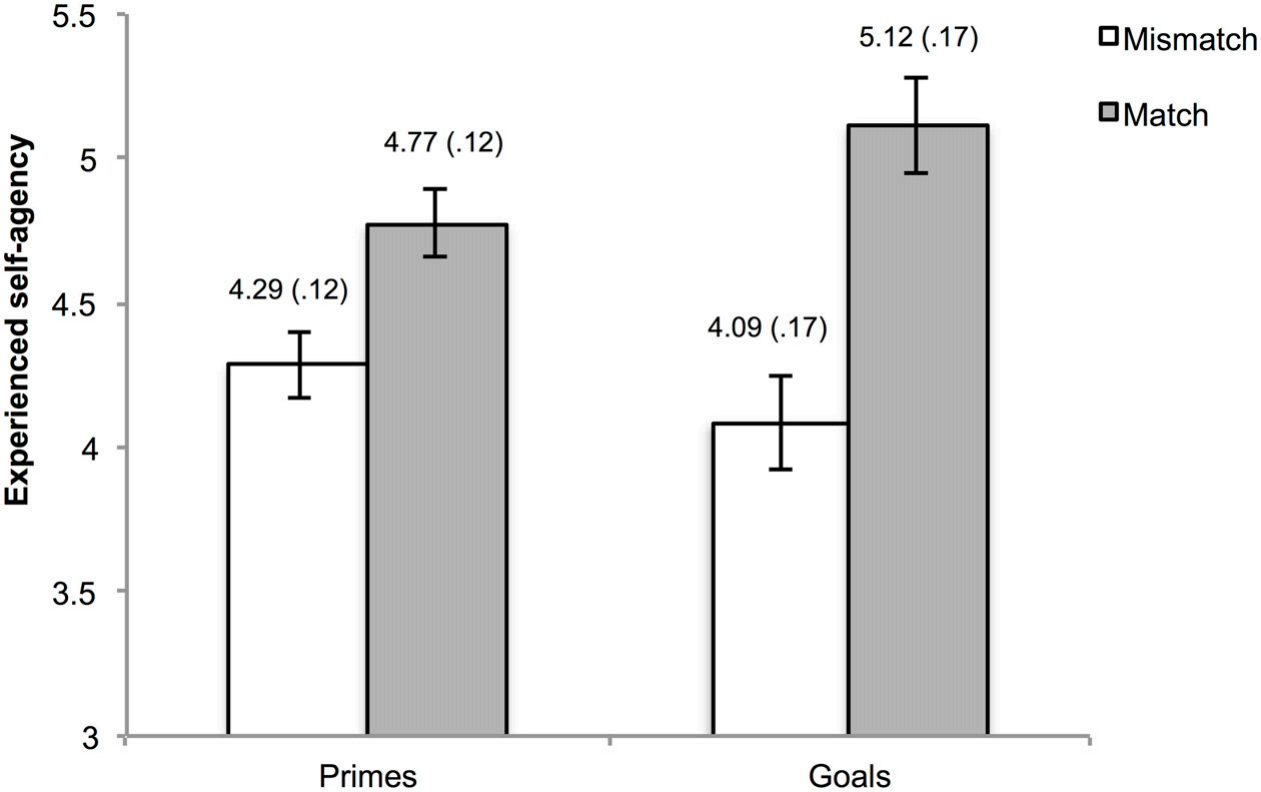
**Reported experience of agency as a function of type of pre-activation and matching**. The numbers above the bars represent the mean and standard error (also reflected by the error bars) of the corresponding condition.

To gain further insight into the interaction, simple main effects using Bonferroni correction (corrected alpha = 0.0125) were calculated. These analyses yielded higher agency ratings for matching vs. mismatching in both the goal, *F*_(1, 29)_ = 11.36, *p* = 0.002, η^2^_ρ_ = 0.28, and outcome priming condition *F*_(1, 29)_ = 10.74, *p* = 0.003, η^2^_ρ_ = 0.27. A marginally significant simple main effect of type of pre-activation was observed within match trials, *F*_(1, 29)_ = 6.66, *p* = 0.02, η^2^_ρ_ = 0.19, but not in mismatch trials, *F*_(1, 29)_ = 2.22, *p* = 0.15, η^2^_ρ_ = 0.07. The means of the cells are depicted in Figure 2.

#### Key-press reaction times

To check whether participants responded differently to the action cue by pressing the key as a function of the type of trial, mean reaction times were submitted to a 2 (type of pre-activation: goal vs. prime) × 2 (matching: mismatch vs. match) repeated measures ANOVA. This analysis yielded a non-significant main effect of type of pre-activation, *F*_(1, 29)_ = 2.68, *p* = 0.11, η^2^_ρ_ = 0.09, indicating no difference in reaction time between goal trials (*M* = 436.25, *SE* = 11.85) and prime trials (*M* = 449.28, *SE* = 9.75). In addition, no difference in reaction time was observed between match (*M* = 445.43, *SE* = 9.77) and mismatch trials (*M* = 440.10, *SE* = 10.69), as evidenced by a non-significant main effect of matching, *F*_(1, 29)_ = 2.38, *p* = 0.13, η^2^_ρ_ = 0.08. Finally, the interaction effect between type of pre-activation and matching was not significant, *F*_(1, 29)_ = 0.05, *p* = 0.83, η^2^_ρ_ = 0.002.

#### Agency rating times

The time participants took to report experienced agency was also assessed by submitting mean rating times (in milliseconds) to a 2 (type of pre-activation: goal vs. primes) × 2 (matching: mismatch vs. match) repeated measures ANOVA. Although the data was non-normally distributed, we refrained from using non-parametric alternatives for previously mentioned reasons. Participants reported experienced agency faster in goal trials (*M* = 1470.69, *SE* = 129.57) than in prime trials (*M* = 1669.35, *SE* = 131.58), *F*_(1, 29)_ = 6.51, *p* = 0.02, η^2^_ρ_ = 0.18. The differences in reaction time between mismatch trials (*M* = 1554.70, *SE* = 122.13) and match trials (*M* = 1585.33, *SE* = 130.79), *F*_(1, 29)_ = 0.49, *p* = 0.49, η^2^_ρ_ = 0.02, as well as the interaction effect between type of pre-activation and matching, *F*_(1, 29)_ = 1.95, *p* = 0.17, η^2^_ρ_ = 0.06, were non-significant.

In short, the behavioral data shows two notable findings. First, participants report higher agency experiences when the observed effect matches vs. mismatches with pre-activated outcome information. This effect tends to be more pronounced in case of goal-based agency inferences than in case of prime-based agency inferences. Moreover, participants provided faster ratings concerning their feeling of agency in goal trials as opposed to prime trials.

### EEG data

#### Data exclusion

Visual data inspection led to the detection of noisy data on one or more channels for five participants. These participants were excluded from further EEG analyses to retain the option of analyzing all 32 × 32 channel pairs. In addition, four participants had no trials left in one or more cells that were created by splitting the data in low and high agency ratings; these participants were also excluded. Hence, the total sample for EEG analysis consisted of 21 participants (*M*_age_ = 21.43, *SD*_age_ = 3.37; 17 females). In addition, 1.67% of the trials were excluded based on semi-automated visual artifact rejection. Finally, trials that were characterized as missing in the agency inference task (i.e., trials in which the key was not pressed within the action interval) were omitted from analyses (2.75%)^2^.

#### Connectivity

**Figure 3:**
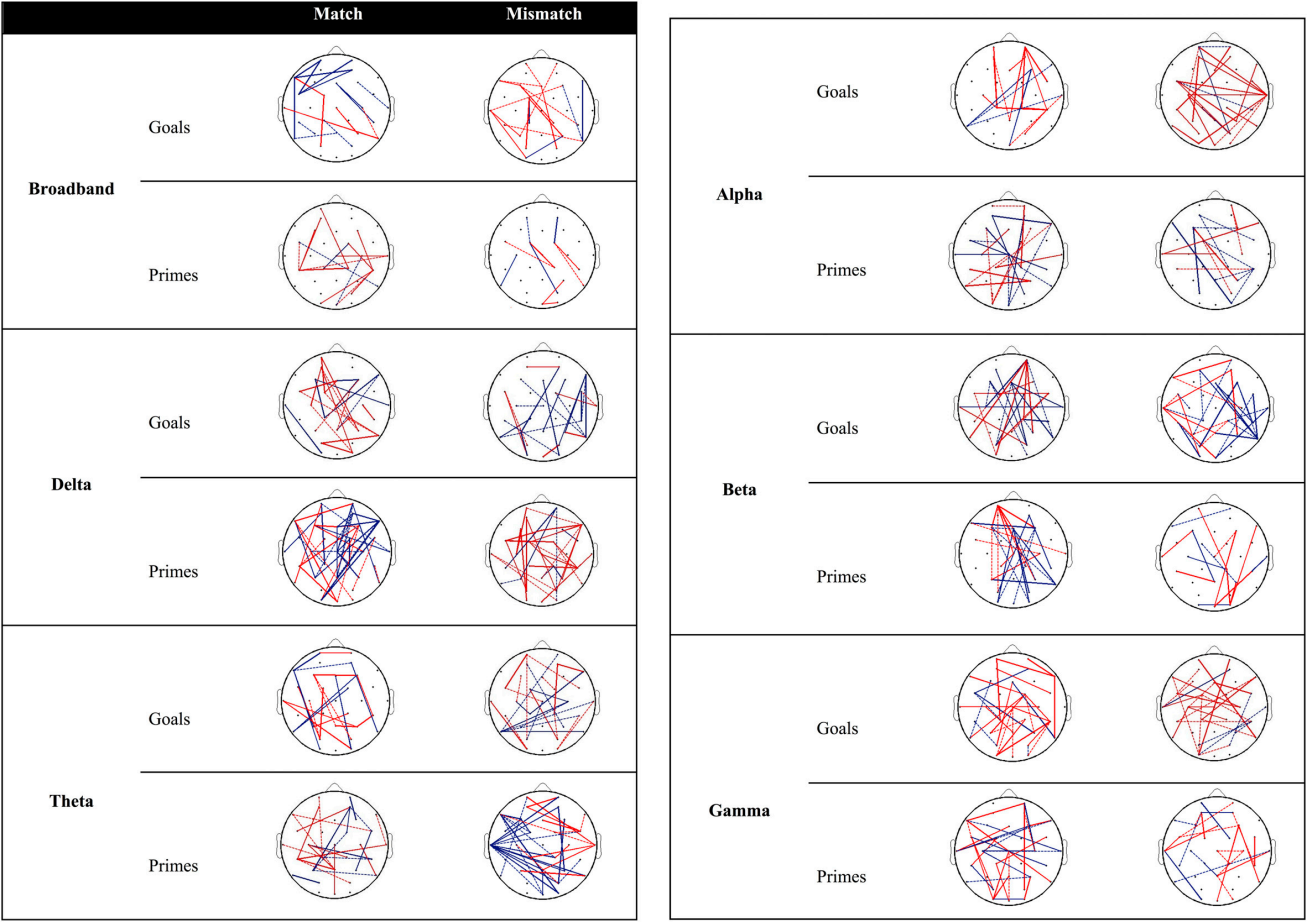
**Significant differences in PLI between low and high agency as a function of matching and type of pre-activation across frequency bands**. Red lines indicate that PLI low agency > PLI high agency, whereas blue lines represents the opposite pattern. Dashed lines represent PLI differences near the significance threshold (0.02 ≤ *p* ≤ 0.03) whereas solid lines represent connections that are more robust across re-runs of the permutation analysis (*p* < 0.02).

Figure 3 provides an overview of connectivity for the contrast between low and high agency as a function of matching and type of pre-activation. Re-running the permutation analyses can result in marginal variation in the null distribution of mean differences. As a result, inclusion of connections with PLI differences near the significance threshold (alpha = 0.05; two-tailed) is subject to similar variation. Dashed lines (0.02 ≤ *p* ≤ 0.03) are used to discriminate these connections from those that are more robust over different runs (i.e., solid lines; *p* < 0.02).

***Goal trials.*** The behavioral data suggests that people experience more self-agency when a goal matches the observed outcome vs. when it does not. In other words, matches are more likely to be associated with high agency, whereas mismatches are associated with low agency^3^. When examining connectivity associated with high agency (vs. low agency) experiences in trials in which goals match with the outcome (Figure 3), increased connectivity is observed between parietal and frontal regions as well as within the frontal cortices in the broadband. With regard to specific frequency bands, high agency experiences during match trials seem particularly governed by increased connectivity in the beta band.

Different connections emerge during low-agency experiences in trials in which goals mismatch with the outcome. Specifically, in the broadband frequency increased connectivity for low agency experiences (compared to high agency experiences) is observed within and between parietal and frontal areas. In addition, this increased connectivity can particularly be observed in alpha and gamma bands.

***Prime trials.*** During experiences of high agency (vs. low agency) in trials in which primes match with the observed effect, increased connectivity can be observed between parietal and frontal regions in the broadband. Increased fronto-parietal connectivity is also present to a larger extent in the delta, theta, alpha, and beta band.

Reports of low agency (as opposed to high agency) during primed mismatch trials are associated with enhanced coupling between parietal and frontal areas. With regard to specific frequency bands, increased connectivity between parietal, and frontal regions during experiences of low agency (vs. high agency) is especially apparent in the delta band.

#### Direction of information flow

The connectivity pattern that was observed in the broadband during high agency experiences in trials in which goals matched action-effects, is in line with previous findings on the neural basis of agency (e.g., Nahab et al., 2011). To explore whether the information flow between the identified nodes is also consistent with prior work (i.e., directed from parietal to frontal lobes), directed phase lag indices were calculated for all conditions in this frequency range (see Table 1 for mean PLI values). As can be seen in Table 2, the signal measured from the left parietal electrode is leading in phase compared to the signal at the left frontal electrode in trials in which goals match the outcome, suggesting that there is a trend of anteriorly directed information flow in these trials. In the other conditions no clear direction of information flow could be observed. It should be noted that the reported effects are not corrected for multiple comparisons. After implementing this correction, none of the dPLI values were different from 0.5 at the conventional significance level of *p* < 0.05.

**Table 1:** **Mean PLI during high (HA) and low experiences of agency (LA) in trials in which (A) goals matched the outcomes, (B) goals mismatched the outcomes, (C) primes matched the outcomes, and (D) primes mismatched the outcomes in broadband frequency**.

**Connection**	**PLI (LA)**		**PLI (HA)**	
	***M***	***SD***	***M***	***SD***
**(A)**				
PO3_FC1	0.18	0.04	0.15	0.03
FC1_F7	0.16	0.04	0.14	0.03
P7_F7	0.12	0.03	0.15	0.03
F7_AF4	0.11	0.04	0.14	0.04
FC5_FP1	0.12	0.03	0.14	0.03
FC5_FP2	0.11	0.03	0.14	0.03
FC5_AF4	0.11	0.04	0.14	0.03
F7_FP1	0.11	0.03	0.14	0.04
CP6_Fz	0.12	0.03	0.15	0.02
**(B)**				
PO4_Fz	0.16	0.04	0.13	0.03
PO3_FC1	0.15	0.04	0.12	0.04
P4_FC1	0.16	0.03	0.13	0.04
CP2_F3	0.16	0.03	0.13	0.04
F3_F7	0.15	0.04	0.12	0.04
P8_F8	0.13	0.03	0.16	0.05
CP1_FC1	0.16	0.03	0.18	0.03
**(C)**				
PO4_CP6	0.16	0.03	0.13	0.03
CP6_FC2	0.17	0.04	0.14	0.03
CP6_F4	0.16	0.05	0.13	0.03
CP2_FP1	0.14	0.04	0.11	0.03
CP2_CP5	0.16	0.03	0.14	0.02
CP5_F3	0.15	0.03	0.13	0.04
CP5_AF3	0.15	0.04	0.12	0.03
CP1_FC2	0.14	0.02	0.16	0.03
**(D)**				
CP2_FC1	0.16	0.03	0.14	0.03
CP6_FC2	0.16	0.04	0.13	0.03
PO4_FC1	0.13	0.03	0.16	0.04
FC2_AF4	0.13	0.03	0.15	0.04

Only connections between parietal and frontal electrodes (based on positions in the international 10/20 system) with robust significant PLI group differences are shown (i.e., solid lines in Figure 3).

**Table 2:** **Results of one-sample *t*-tests for dPLI values during high (HA) and low experiences of agency (LA) in trials in which (A) goals matched the outcomes, (B) goals mismatched the outcomes, (C) primes matched the outcomes, and (D) primes mismatched the outcomes in broadband frequency**.

**Connection**	***t***	***p***	**Direction effect**
**(A)**			
LA_PO3_FC1	−0.21	0.83	–
LA_FC1_F7	−0.65	0.53	–
HA_P7_F7	2.46	0.02	↑
HA_F7_AF4	2.11	0.05	↑
HA_FC5_FP1	3.28	0.004	↑
HA_FC5_FP2	3.23	0.004	↑
HA_FC5_AF4	1.40	0.18	–
HA_F7_FP1	2.74	0.01	↑
HA_CP6_Fz	−1.09	0.29	–
**(B)**			
LA_PO4_Fz	−0.04	0.97	–
LA_PO3_FC1	−0.59	0.56	–
LA_P4_FC1	0.30	0.77	–
LA_CP2_F3	−0.13	0.90	–
LA_F3_F7	−0.81	0.43	–
HA_P8_F8	1.73	0.099	↑
HA_CP1_FC1	0.37	0.72	–
**(C)**			
LA_PO4_CP6	−0.53	0.60	–
LA_CP6_FC2	−2.89	0.009	↓
LA_CP6_F4	−2.25	0.04	↓
LA_CP2_FP1	−1.91	0.07	↓
LA_CP2_CP5	2.02	0.06	↑
LA_CP5_F3	−0.89	0.38	–
LA_CP5_AF3	0.15	0.88	–
HA_CP1_FC2	0.62	0.54	–
**(D)**			
LA_CP2_FC1	−0.35	0.73	–
LA_CP6_FC2	−1.40	0.18	–
HA_PO4_FC1	0.09	0.93	–
HA_FC2_AF4	−1.16	0.26	–

Only connections between parietal and frontal electrodes (based on positions in the international 10/20 system) with robust significant PLI group differences are shown (i.e., solid lines in Figure 3). The arrows represent the direction of the information flow between the two nodes (i.e., electrodes) specified in the contrast. Upward arrows indicate that the first node is sending information to the second node, whereas downward arrows indicate that the first node is receiving information of the second node. Arrows are presented for p-values < 0.10.

## Discussion

Building on recent interest in neural networks underlying agency processing (David, 2012), the present study examined cortical information flow during inferences of agency. Whereas some insights into the networks underlying agency processing have been provided by previous studies employing fMRI (David et al., 2007; Nahab et al., 2011), here we offered a first attempt to investigate this connectivity by tapping into the mechanism that is proposed to underlie neural communication (i.e., phase synchronization).

The role of inference processes in self-agency experiences is supported by the current behavioral data. In line with the inference model (Wegner, 2002) and previous work (Wegner and Wheatley, 1999; Aarts et al., 2005; Van der Weiden et al., 2011, 2013), participants reported higher agency experiences when pre-activated knowledge was congruent vs. incongruent with actual outcomes. Importantly, these results cannot be easily accounted for by the comparator account, as predictive motor processes were unreliable (or even absent) due to the experimental set-up. Specifically, there was no causal relation between the key press of participants and the presentation of the outcome, which restricts the motor system in its prediction of sensory action consequences (Sato, 2009). Accordingly, the reported experiences of agency are likely to be informed by cognitive inferences formed upon the occurrence of the outcome.

The results of the EEG data provide insight into neural connectivity underlying agency inferences during matches and mismatches. Increased coupling between parietal and frontal cortices, as well as within frontal areas, was identified in the broadband during high agency experiences in trials in which outcomes matched prior goals. These regions have been associated with agency processing in general (David et al., 2008; Sperduti et al., 2011) and agency inferences in particular (Renes et al., 2013). The PPC has been implicated in the detection of congruence between motor predictions and sensory action consequences, and has mainly been activated during mismatches (David, 2010). Nevertheless, Renes et al. (2013) have also identified activity in this region during matches, suggesting that it might be involved in more general comparative processes between outcome expectations and action-effects. Activity in prefrontal areas has been linked to a conscious monitoring function (i.e., the conscious experience of having caused an outcome or not; David, 2010). Although the observed fronto-parietal connectivity concurs with this prior research, it is important to note that observed connectivity during agency inferences was not restricted to these areas, as can be seen in Figure 3.

Connectivity between parietal and frontal areas in the broadband was also observed during low agency experiences in trials in which goals mismatched with the outcome. Notably, however, the coupling within frontal areas that was observed during high agency in match trials was not detected during low agency experiences in mismatch trials. A possible explanation for this finding is that this frontal network is especially involved in the ascription of outcomes to oneself as opposed to external sources. Some indirect support for this idea comes from research on self-referential processing showing increased activity of the medial prefrontal cortex when participants judged personality traits as self-descriptive vs. not self-relevant (Moran et al., 2006; Rameson et al., 2010). This fits with our observation that the frontal network was not involved in case of mismatching outcomes that were not ascribed to oneself (i.e., that were deemed to be non-relevant).

Beyond the mere presence of increased coupling, a trend of directionality pointing toward information flow from parietal to frontal cortices was observed in the broadband during high agency experiences following from outcomes matching goals. This finding is in line with results by Nahab et al. (2011) who speculated that the PPC serves as a low-level congruence detection network that transmits mismatch information to prefrontal cortices in order to give rise to higher order agency processing (i.e., a conscious experience of agency). Although this observation is exciting, it is important to note that the observed directionality in the current study was relatively weak (in terms of statistical significance) and absent in trials in which goals mismatched the actual outcome. That is, whereas increasing coupling between parietal and frontal regions was observed during low agency experiences in these trials, parietal nodes were not leading in phase compared to frontal nodes. Therefore, interpretations with regard to direction of information flow should be made with caution. More generally, it is important to note that there is no unique relationship between the time series recorded by EEG and their underlying source, allowing only crude interpretations concerning underlying brain regions. Importantly, however, the main interest of the present study was to elucidate connectivity between frontal and more posterior parts of the brain, rather than to relate specific brain areas to agency inferences.

In addition to neural communication between cortical regions in the broadband frequency, interactions in specific frequency bands were assessed. Intriguingly, fronto-parietal connections were present across frequency bands, while none of the bands seemed particularly involved in agency inferences as a whole. These observations might be attributable to the complex nature of agency processing, in the sense that it encompasses functions that have been related to specific bands, such as keeping outcome representations active in working memory (associated with theta band oscillations; Klimesch et al., 2005) and, in the case of goal-based inferences, the prioritizing of top-down influence (i.e., goals) over novel events (associated with beta band oscillations; Engel and Fries, 2010). Accordingly, the observed connectivity in the variety of bands might be a reflection of the different dimensions of the integration process involved in agency inferences (Varela et al., 2001).

Recent findings suggest that agency experiences can result from goal-based inferences as well as from primed-based inferences (Van der Weiden et al., 2013). Based on these findings, we examined the neural communication involved in both type of agency inferences. When comparing connectivity patterns between goals and primes in the broadband, frontal connections were observed during high agency experiences in trials in which goals matched the outcome that were absent in trials in which primes matched the outcome. Similarly, more fronto-parietal coupling was observed in goal trials than in prime trials during low agency experiences in mismatch trials. This general decrease in connectivity associated with primes (vs. goals) might be explained by differences in the process underlying the two types of pre-activation (Van der Weiden et al., 2013). In contrast to goals, mere priming of outcome information is not assumed to install an attentional control process that maintains the specific outcome active in mind, while inhibiting other irrelevant (but associated) items at hand. Therefore, the activation of the outcome representation by priming (compared to goals) might be more transient and less stable. The behavioral data provides evidence for this notion. First, the difference in agency experiences resulting from matches and mismatches tends to be more predominantly expressed in goal trials than in prime trials. In addition, participants were significantly faster to report experienced self-agency in the former (vs. the latter) trials. These findings are in line with the notion that agency inferences occurring via priming processes are less stable and noisier than goal-based inferences, which may account for the reduced connectivity associated with the former processes.

This line of reasoning might shed light onto the recent observation that patients suffering from schizophrenia show specific disturbances in prime-based inferences processes whereas their goal-based inferences seem intact (Renes et al., 2013). Schizophrenia has been related to reduced structural connectivity between various brain regions, including reduced integrity of white matter tracts connecting parietal and frontal nodes (Ellison-Wright and Bullmore, 2009; Voineskos et al., 2010; Whitford et al., 2011). Given that anatomical connections restrict the functional networks that can be formed (Fries, 2005), agency inferences that rest on fronto-parietal functional connectivity are likely to be disturbed as well. The present study suggests that functional connectivity related to prime-based inferences is weaker compared to goal-based inferences. When taking into account that only the prime-driven processes are disturbed in schizophrenia patients, it can be speculated that the relatively strong functional connectivity pattern underlying inferences based on goals, might allow schizophrenic patients to experience agency despite decreased anatomical fronto-parietal connectivity. In contrast, inferences based on primes are already associated with weaker functional connectivity and accordingly might not be able to overcome these structural abnormalities. However, the notion that primed-based agency inferences are reduced in schizophrenic patients as a result of the quality of fronto-parietal anatomical connectivity awaits further testing.

There are several methodological limitations that warrant consideration when interpreting the present results. By examining connectivity on the scalp we cannot exclude the possibility that observed differences between conditions have been affected by spontaneous and systematic changes of distant sources. That is, due to the discontinuity PLI, noise induced by these sources can shift phase leads to phase lags, which in turn might give rise to spurious differences or, oppositely, mask real differences in connectivity (Vinck et al., 2011). Future studies incorporating source-localization procedures might provide additional insight into the influence of distant sources. Another factor that might affect PLI measurements is the number of trials used to estimate this index. When this number is small, as in the current study, PLI values tend to be overestimated, especially in case of small PLI values (Vinck et al., 2011). Note, however, that this overestimation of PLI would be expected in both low and high agency conditions. As such, the connectivity difference of interest is relatively unaffected by this issue. A final confounding factor in the present study is the multiple comparisons problem. Statistical analysis of EEG data inherently copes with testing of condition effects at a large number of pairs, across multiple frequency bands. Although there are methods to correct for multiple testing, these methods are either overly conservative when a large number of tests is conducted, or focused on networks rather than individual connections. Given the exploratory aim of the present research, an uncorrected comprehensive overview of connectivity is provided. Accordingly, observed connectivity has to be interpreted with some caution.

## Conclusion

To conclude, we have demonstrated the potential of recent methodological advances in the quantification of brain dynamics to elucidate the neural basis underlying inferences of agency. In particular, we were able to extend prior research that has mainly focused on localized activation and provide preliminary support for the existence of fronto-parietal interactions involved in sending information from parietal to frontal areas to arrive at the conscious experience of agency. By doing so, we hope that the present results will encourage future research to move beyond mere snapshots of the brain and to further explore the neural networks underlying agentive self-awareness.

## Author note

The work in this paper was supported by a VICI-grant 453-10-003 from the Dutch Organization for Scientific Research.

### Conflict of interest statement

The authors declare that the research was conducted in the absence of any commercial or financial relationships that could be construed as a potential conflict of interest.

## Footnotes

^1^The authors are aware that the within-subject nature of the data violates the exchangeability assumption of the permutation tests and thus increases the likelihood of false positives. However, given that the design of the study leads to varying numbers of trials in the low and high agency condition (precluding within-subject permutation), as well as our aspiration to visualize networks, this analytical procedure was deemed most appropriate. Nevertheless, caution is advised when interpreting the results.

^2^Although the analyses of behavioral data and EEG data differ (in the sense that different contrasts are assessed), we checked whether the reported findings for behavioral data change when excluding the artifact trials and participants that are excluded from the EEG analysis. The repeated measures ANOVA yielded a main effect of matching, *F*_(1, 20)_ = 8.36, *p* = 0.009, η^2^_ρ_ = 0.29, showing that matching outcomes corresponded with higher agency experiences than mismatching outcomes. The main effect of type of pre-activation, *F*_(1, 20)_ = 0.02, *p* = 0.89, η^2^_ρ_ = 0.001 and the interaction between type of pre-activation and matching, *F*_(1, 20)_ = 1.27, *p* = 0.27, η^2^_ρ_ = 0.06 were not significant.

^3^Note that the behavioral data indicates that participants sometimes report low agency in a match trial and high agency in a mismatch trial. These experiences, and corresponding connectivity, are not likely to reflect agency processes emerging from prime-based or goal-based inferences—and are thus not explicitly discussed in the result section.
