# Supplementary material for: Cortical information flow during inferences of agency
Source: Front Hum Neurosci. 2014 Aug 14;8:609. doi: 10.3389/fnhum.2014.00609 (PMC4132368; doi:10.3389/fnhum.2014.00609)
Supplement: Supplementary file 1 [file Image1.PDF]

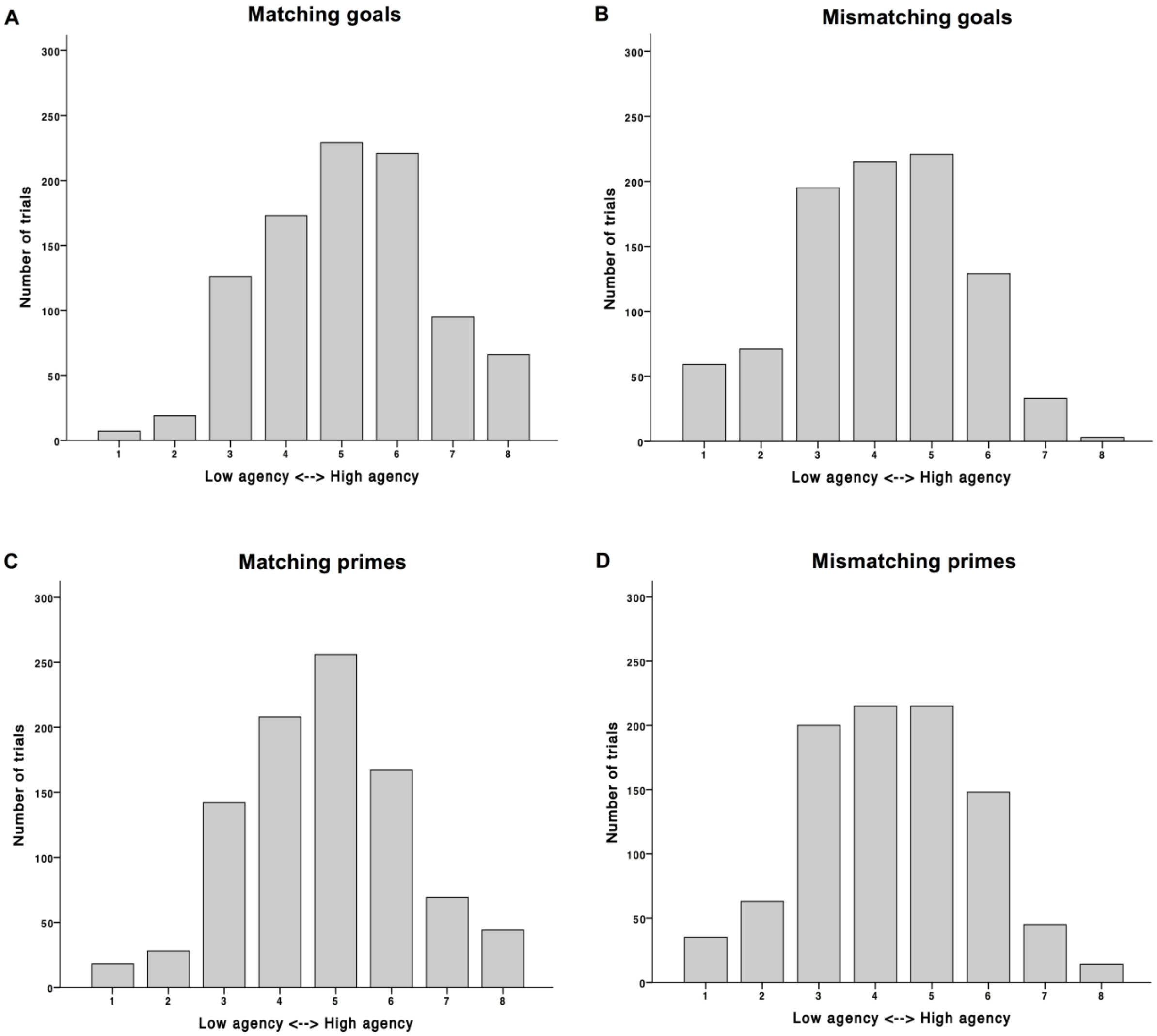

Figure S1:

Frequency distribution of self-agency ratings in trials in which (A) goals matched the outcome, (B) goals mismatched the outcome, (C) primes matched the outcome and (D) primes mismatched the outcome. In the goal condition, high agency ratings were reported on 65.3% of the match trials and on 41.7% of the mismatch trials, whereas in the prime condition high agency was reported on 57.5% of the match trials and on 45.1% of the mismatch trials.
